# Reduced model-based decision-making in gambling disorder

**DOI:** 10.1038/s41598-019-56161-z

**Published:** 2019-12-23

**Authors:** Florent Wyckmans, A. Ross Otto, Miriam Sebold, Nathaniel Daw, Antoine Bechara, Mélanie Saeremans, Charles Kornreich, Armand Chatard, Nemat Jaafari, Xavier Noël

**Affiliations:** 1 Psychological Medicine Laboratory, Université Libre de Bruxelles, Brussels, Belgium; 2 Department of Psychology, McGill University, Montréal, Canada; 3 Department of Psychiatry and Psychotherapy, Charité Universitätsmedizin Berlin, Berlin, Germany; 4 Department for Social and Preventive Medicine, University of Potsdam, Potsdam, Germany; 5 Princeton Neuroscience Institute, Princeton University, Princeton, NJ 08540 USA; 6 Department of Psychology, Princeton University, Princeton, NJ 08540 USA; 7 Department of Psychology, University of Southern California, Los Angeles, California USA; 8 Psychiatric Institute, Universitary Hospital Brugmann, Brussels, Belgium; 9 Faculty of Psychology, University of Poitiers, Poitiers, France; 10 Faculty of Medicine, University of Poitiers, Poitiers, France

**Keywords:** Human behaviour, Learning algorithms

## Abstract

Compulsive behaviors (e.g., addiction) can be viewed as an aberrant decision process where inflexible reactions automatically evoked by stimuli (habit) take control over decision making to the detriment of a more flexible (goal-oriented) behavioral learning system. These behaviors are thought to arise from learning algorithms known as “model-based” and “model-free” reinforcement learning. Gambling disorder, a form of addiction without the confound of neurotoxic effects of drugs, showed impaired goal-directed control but the way in which problem gamblers (PG) orchestrate model-based and model-free strategies has not been evaluated. Forty-nine PG and 33 healthy participants (CP) completed a two-step sequential choice task for which model-based and model-free learning have distinct and identifiable trial-by-trial learning signatures. The influence of common psychopathological comorbidities on those two forms of learning were investigated. PG showed impaired model-based learning, particularly after unrewarded outcomes. In addition, PG exhibited faster reaction times than CP following unrewarded decisions. Troubled mood, higher impulsivity (i.e., positive and negative urgency) and current and chronic stress reported via questionnaires did not account for those results. These findings demonstrate specific reinforcement learning and decision-making deficits in behavioral addiction that advances our understanding and may be important dimensions for designing effective interventions.

## Introduction

Modern theories of addictive behaviors are built on basic neural and cognitive decision mechanisms, and posit an imbalance between past-oriented habits (e.g., drinking alcohol automatically in a given context) and present and future-oriented goals (e.g., limiting alcohol use), thus resulting in a lack of consideration for the consequences of the actions^1–3^. Deficits in goal-directed learning and control **(**e.g., prepotent response inhibition, set-shifting) have been observed across a range of disorders characterized by compulsivity such as addiction^4–6^ and obsessive-compulsive disorder^7,8^. The case of gambling disorder (GD) is of particular interest. Recently reclassified alongside substance use disorder^9^, mainly because those syndromes share clinical (e.g., craving, escalation in use) and neurobiological (e.g., abnormal fronto-striatal network) characteristics^10,11^, GD offers the opportunity to understand addiction without potentially confounding neurotoxicity associated with acute or chronic use of psychoactive substance^12^.

For effort and energy saving^13,14^, adaptive choice behavior relies on optimal orchestration between two forms of instrumental decision systems: the goal-directed system learns about the contingency between actions and outcomes and ensures that behavior is appropriate given our motivational state and/or desire for these outcomes, while the ‘habitual’ system enables actions that has been trained or ‘stamped in’ to the extent that these actions become stimulus- rather than goal-driven^15^. The way in which those systems interact in healthy and psychopathological conditions have received considerable attention in recent years^16–19^.

Whether compulsive behaviors are automatically driven by contextual elements without outcome expectations (i.e., habit)^20,21^ or if they remain mainly goal-oriented^22–24^ or both^25^ is still debated^26^. While animal model studies employing outcome-devaluation techniques find that agents presenting persistent drug use are measurably less sensitive to devaluation^27^, particularly in those with higher trait impulsivity^28^, human studies in individuals with substance use disorder (SUD) have yielded mixed results^23,29,30^.

As an attempt to better characterize those two forms of learning that remain difficult to dissociate experimentally^26^, habit and goal-directed control has recently been computationally formalized as ‘model-free’ (MF) and ‘model-based’ (MB) reinforcement learning (RL). Crucially MF and MB learning can be disentangled by using sequential decision-making paradigms. Critically, a measure of individuals’ utilization of MB RL (on a sequential decision task, the two-step task) correlates with sensitivity to outcome-devaluation paradigms classically used to probe the balance between goal-directed and habitual control^31^. In accordance with predictions based on animal studies^32^, impaired MB RL has been linked to a wide range of compulsive symptoms^19,33,34^.

Research investigating the relative contribution of MB and MF in clinical populations with substance use disorder (SUD) has provided a less consistent picture. While individuals with alcohol use disorder sometimes showed impaired MB after negative outcome (e.g., a non-rewarded trial)^35^, other studies find no difference in expression of MB choice between alcoholic and non-alcoholic participants^18,33,36^. Whereas binge drinkers had impaired goal-directed behavior in a computational two-step sequential decision-making task^37^, no association of goal-directed or habitual control and alcohol intake was found in young social drinkers^38^, neither between children of alcoholic father compared to their controls^39^. However, impaired MB was found in methamphetamine-dependent subjects^33^. The impact of impaired decision-making on alcohol relapse has been recently clarified in a large sample of detoxified individuals with alcohol dependence^36^. The risk of relapse during a follow-up period of 48 weeks was magnified in subjects holding high alcohol expectations together with low model-based control. However, reduced model-based control per se was not associated with subsequent relapse.

Critically, the discrepancy between studies in substance dependent individuals suggest that some, but not all substances of abuse have a transient or lasting deleterious impact on the balance between model-free and model-based control. If this was the case, the imbalance between both systems would not necessary be a transdiagnostic marker for compulsive disorders, as previously suggested^19,40^. One approach to clarify whether the balance between model-free and model-based control serves as a transdiagnostic marker or is instead a consequence of certain drugs intake is to study behavioral, non-substance addiction (e.g., gambling use disorder). Indeed, focusing on active PG therefore allows to directly evaluate addiction’s impact on decision-making, while removing the substance’s neurotoxicity as a confounding factor^12^. Moreover, beyond the clinical similarities between GD and SUD, shared brain vulnerability markers relevant for the study of the habitual and goal-directed modes of action have been found across gambling and substance-used disorders. For instance, hyperdopaminergic activity was found in the dorsal striatum in gamblers and substance abusers, a region implicated to habit-based responding^11^ associated with GD symptom severity^41^.

Although some deficiencies in the executive functioning have been reported in subjects with GD^42^, the extent to which goal-directed (versus habitual) learning might be impaired in subjects with GD remains unexplored. Thus, finding its justification from clinical and neurobiological data, the present study sought to ascertain whether problem gamblers have impaired MF/MB orchestration on a two-step task^43^. A previous study^35^ reported impaired goal-directed strategy specifically after non-rewarded trials in individuals with alcohol addiction. Moreover, reduced loss aversion in GD and alcohol dependence^44^ suggest that both addictive disorders show reduced sensitivity towards negative outcomes. In GD, this matches well with the clinical phenomenon of loss chasing, where GD patients continue to gamble after severe losses^45^. To further elucidate these mechanisms, we tested how outcome valence would differentially impact reinforcement learning in GD.

## Results

### Sample characteristics

Our sample consisted of 82 participants, 33 CP and 49 PG. Our final sample consisted of 78 participants: 45 PG and 33 CP. Table 1 depicts the demographic and clinical variables of PG and CP as well as between-groups comparisons.

**Table 1:** Demographic and psychological measures for Problem Gamblers (PG) and Control Participants (CP): mean (SD).

Variable	PG	CP	Between groups difference
Gender ratio (men/female)	38/7	29/4	X²(1) = 0.19, p = 0.67
Age	31.31 (9.11)	31.27 (7.93)	t(76) = 0.02, p = 0.98
Years of education	12.73 (2.63)	12.88 (2.87)	t(76) = 0.23, p = 0.82
OSPAN	74.42 (10.96)	79.19 (11.40)	t(76) = 1.97, p = 0.07
CPGI	14.13 (5.02)	0	t(44) = 18.88, p < 0.001***
DSM-V	6 (1.38)	0	t(44) = 29.13, p < 0.001***
Impulsivity (UPPS-P)	49.58 (9. 5)	48.12 (6.16)	t(75.02) = 0.82, p = 0.42
*Negative Urgency*	10.82 (2.97)	8.97 (2.14)	U = 453.5, p < 0.01**
*Positive Urgency*	12.02 (2.18)	10.45 (2.03)	t(76) = 3.23, p < 0.01**
*Lack of premeditation*	8.33 (3.05)	8.61 (2.03)	U = 645.5, p = 0.32
*Lack of perseverance*	7.51 (3.19)	8.33 (2.34)	U = 577, p = 0.09
*Sensation seeking*	10.89 (3.13)	11.76 (2.28)	t(76) = 1.35, p = 0.18
SCL-90-R	70.13 (47.14)	40.85 (29.37)	U = 465.5, p < 0.001***
Audit	9.22 (8.36)	10.48 (6.1)	U = 600, p = 0.15
Smoker | Non-smoker	20/25	15/18	X²(1) = 0.01, p = 0.93
FTND	4.95 (2.61)	3.47 (2.61)	t(33) = 1.66, p = 0.11
Beck Depression Inventory	7.44 (5.79)	4.21 (3.94)	U = 488, p = 0.01**
Negative affect	22.2 (9.3)	18.3 (5.75)	U = 580.5, p = 0.1
STAI-YA	37.51 (12.56)	33.3 (9.11)	U = 611.5, p = 0.19
STAI-YB	44.98 (12.56)	39.36 (10.27)	t(76) = 2.05, p < 0.05*
SRRS	289 (183.28)	222.83 (221.14)	U = 430, p < 0.05*
Current stress intensity	3.46 (2.94)	2.75 (2.19)	U = 575, p = 0.13

Significative differences between groups are displayed in bold. All the tests are performed with a two-tailed Student t-test, Mann-Whitney U or a Chi-square test. Welch correction was applied to Student t-tests when Levene’s test for homogeneity of variances was significant (p < 0.05).

### Analyses of choice behavior

**Figure 1:**
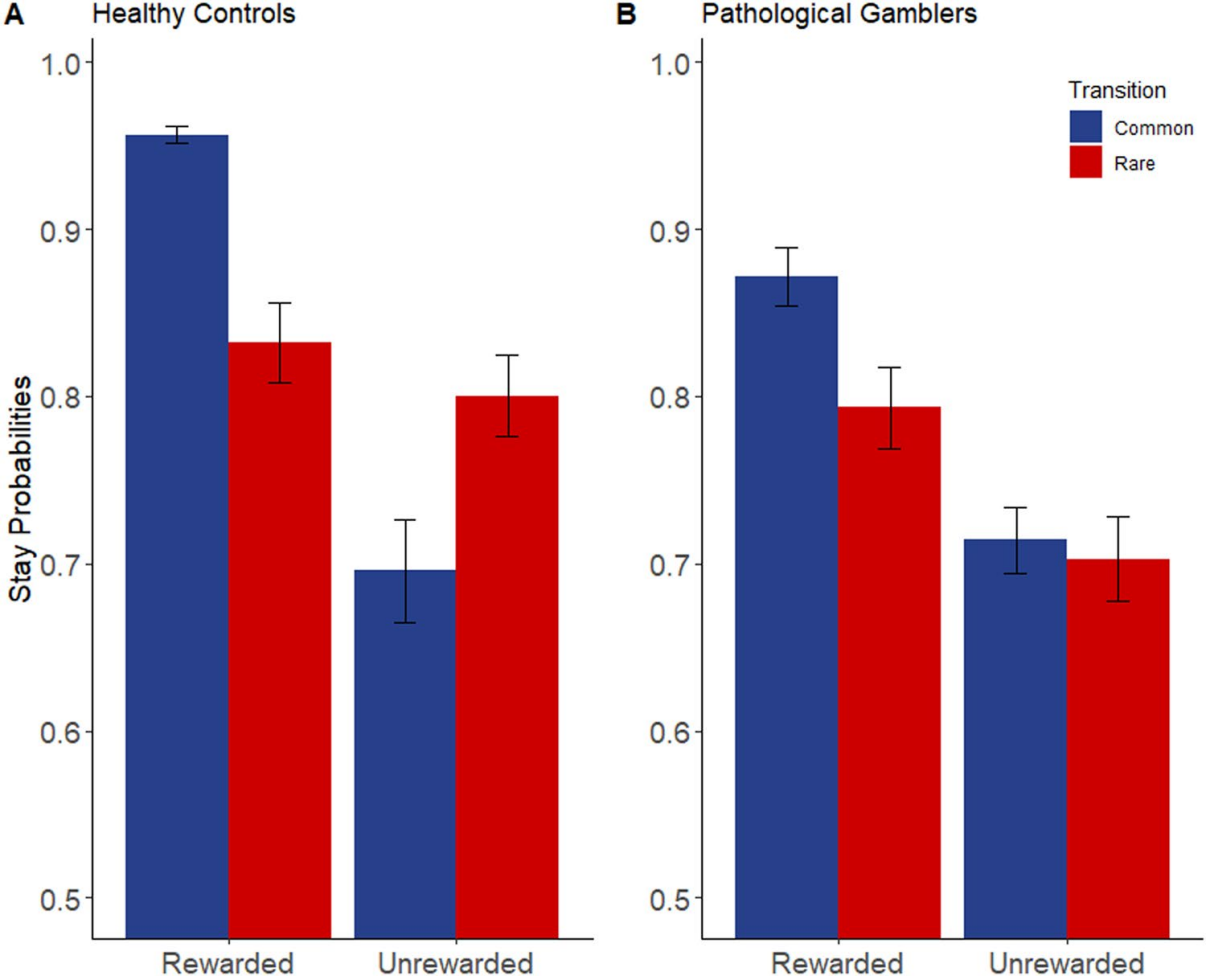
Probabilities to maintain the previous first stage choice depending on the transition and the reward during the previous trial among (**A**) healthy subjects and (**B**) pathological gamblers. Error bars represent two times the standard error.

The regression analysis (see Table 2 and Fig. 1) confirmed the basic signatures of MF and MB strategies, expressed as significant effects of both previous outcome (MF learning; β (SE) = 0.55 (0.06), p < 0.001) and the interaction between previous outcome and transition type (MB learning; β (SE) = 0.32 (0.06), p < 0.001). Moreover, the regression revealed that PG and CP did not appear to differ in their MF choice contributions, as evidenced by the absence of a significant two-way group and previous outcome interaction (p = 0.67). Critically, we observed a significant three-way interaction between group (PG versus CP), previous outcome, and previous transition type (β (SE) = −0.12 (0.06), p < 0.05), indicating an attenuated MB learning signature in PG. As the MB strategy is the optimal reward-harvesting strategy in this task, we found that the proportion of rewarded trials differed significantly between the two groups, whereby the CP group was rewarded significantly more often (mean reward rate: 57.31%) than the PG group (mean reward rate: 54.83%) (F(1,78) = 7.23, p < 0.01, ƞ^2^_p_ = 0.09).

**Table 2:** Logistic regression coefficients indicating the influence of previous trial’s outcome, previous trial’s transition, and group on response repetition.

Coefficient	Estimate (SE)	z value	P value
(Intercept)	1.67 (0.1)	16.26	<0.001***
Group	−0.16 (0.1)	−1.54	0.12
Outcome	0.55 (0.06)	9.05	<0.001***
Transition	0.2 (0.05)	3.86	<0.001***
Group * Outcome	−0.02 (0.06)	−0.42	0.67
Group * Transition	0.04 (0.05)	0.7	0.48
Outcome * Transition	0.32 (0.06)	5.14	<0.001***
Group * Outcome * Transition	−0.12 (0.06)	−2	<0.05**

Significant results are displayed in bold. *Significance at the 0.05 level; **Significance at the 0.01 level; ***Significance at the 0.001 level.

Secondly, logistic regressions which separately examined previously rewarded and unrewarded trials (see Table 3) revealed that in both cases, the entire population expressed a basic MB effect (expressed as a main significant effect of transition; rewarded trials (β (SE) = −0.12 (0.05), p < 0.05); unrewarded trials (β (SE) = 0.51 (0.1), p < 0.001)). More importantly, this MB estimate was significantly lowered in PG only after a negative outcome, as shown by a significant negative group * previous transition interaction (β (SE) = 0.16 (0.05), p < 0.01) after a negative outcome but not after a positive outcome (β (SE) = −0.09 (0.1), p = 0.36).

**Table 3:** Logistic regression coefficients indicating the influence of previous trial’s transition and group on response repetition depending on the previous trial’s outcome.

Coefficient	Unrewarded previous trial			Rewarded previous trial		
	Estimate (SE)	z value	P value	Estimate (SE)	z value	P value
(Intercept)	1.12 (0.09)	11.93	<0.001***	2.22 (0.14)	16.07	<0.001***
Group	−0.13 (0.09)	−1.41	0.16	−0.18 (0.14)	−1.34	0.18
Transition	−0.12 (0.05)	−2.16	<0.05*	0.51 (0.1)	5.32	<0.001***
Group * Transition	0.16 (0.05)	2.95	<0.01**	−0.09 (0.1)	−0.92	0.36

Significant results are displayed in bold. * Significance at the 0.05 level; ** Significance at the 0.01 level; *** Significance at the 0.001 level.

### Response time (RT) analyses

**Figure 2:**
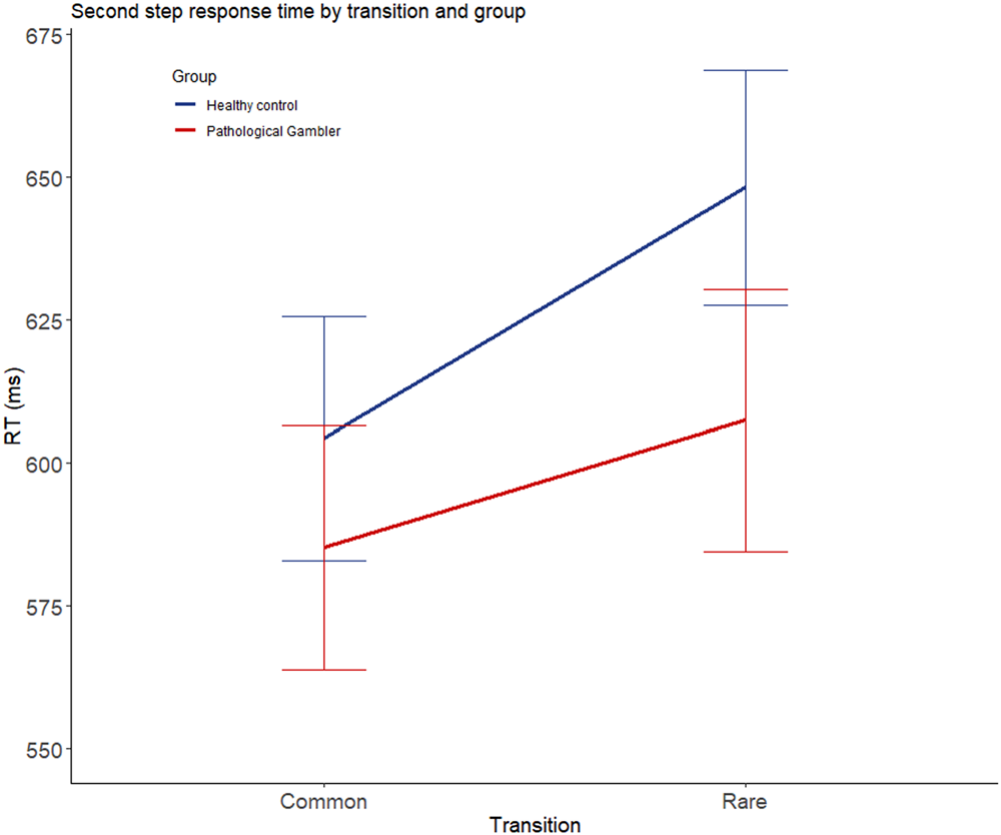
Reaction time in millisecond depending on the transition among both groups. The error bars represent the standard error.

In the mixed ANOVA comparing the second step’s response time according to the transition between both groups (see Fig. 2), a main significant effect of the transition was found (F(1,76) = 43.72, p**<**0.001, η^2^_p_ = 0.37). The participants are slower when the trial’s transition was rare (M = 624.74 ms, SD = 140.61 ms) than common (M = 593.33 ms, SD = 134.15 ms). The interaction between transition type and group also achieved significance (F(1,76) = 4.64, p = 0.03, η^2^_p_ = 0.06). Post-hoc analyses shows that the RT difference between common and rare transition is significantly higher (t(76) = 2.15, p = 0.03, d_s_ = 0.49) in CP (M = 43.82 ms, SD = 45 ms) than in PG (M = 22.29 ms, SD = 42.61 ms).

**Figure 3:**
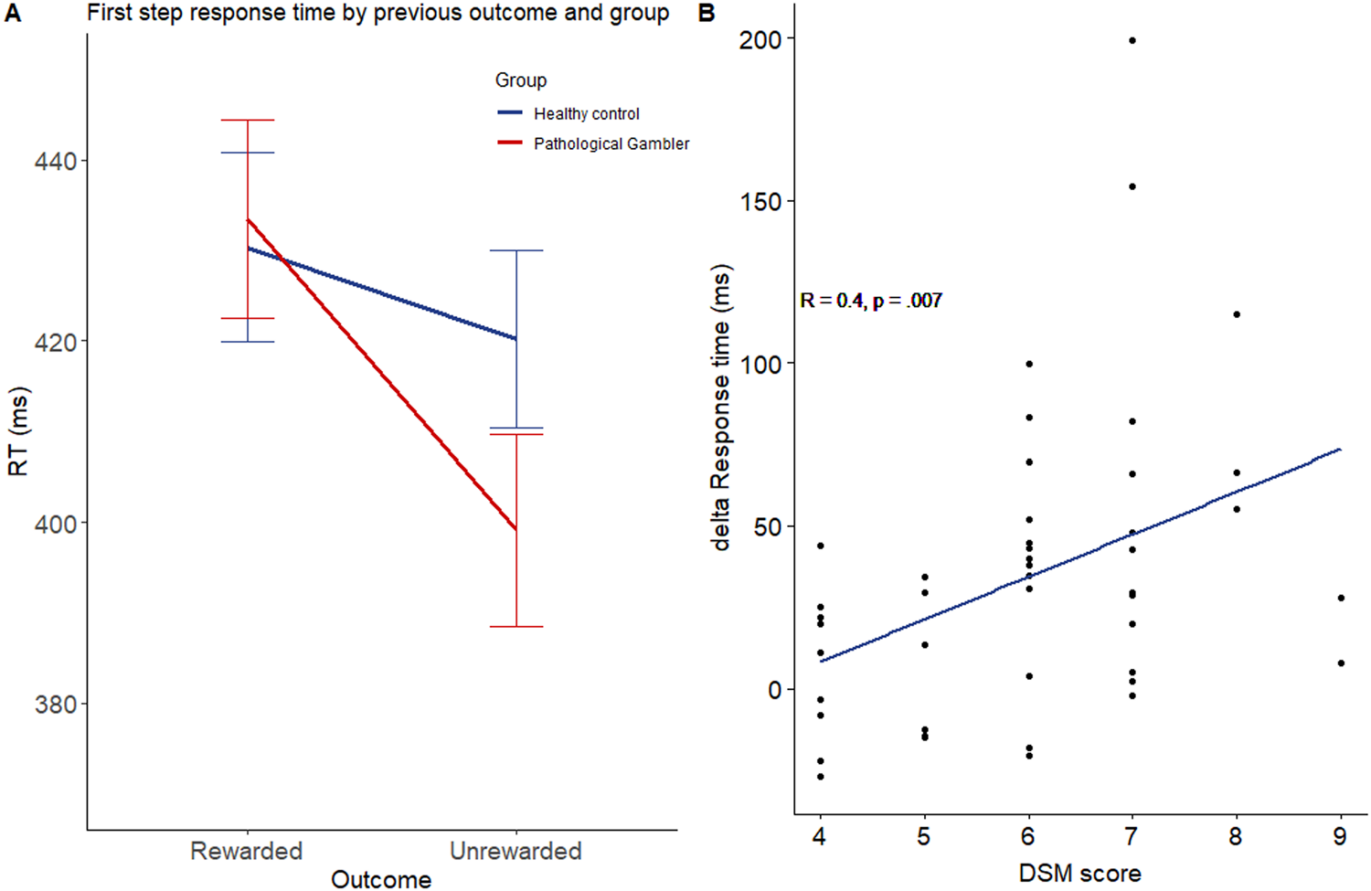
(**A**) Reaction time in millisecond depending on the previous trial outcome among both groups. (**B**) Correlation among PG between gambling severity measured by the DSM score and the response time acceleration after a negative outcome. The error bars represent the standard error.

Based on past finding that MB control is associated with slower reaction times than MF^46^ and because we found that PG had MB deficit after an unrewarded trial, we also examined if the previous losses resulted in faster next trial RTs in PG compared to CP. We used a second mixed ANOVA to analyze the effect of the previous trial’s outcome on the first-choice response time in both groups. A significant main effect of the outcome was found (F(1,76) = 23.3, p < 0.001, η^2^_p_ = 0.24), indicating that participants were faster to make first-stage choices after an absence of reward (M = 406.23 ms, SD = 84.18) than after a positive outcome (M = 432.14 ms, SD = 93.3). The interaction between outcome and group (F(1,76) = 6.92, p = 0.01, η^2^_p_ = 0.08) also achieved significance (see Fig. 3A). Post-hoc analyses revealed that PG group made first-stage choices more quickly (t(44) = 5.45, p < 0.001, d_z_ = 0.82) after a negative outcome (M = 396.53 ms, SD = 89.78) than after a positive outcome (M = 433.47 ms, SD = 102.69). This differential pattern of RTs was not observed in the CP group (t(32) = 1.57, p = 0.13). Interestingly, the difference in response time in PG between rewarded and unrewarded trials significantly correlated with the number of symptoms for GD based on the DSM-V^47^ (r = 0.40, p = 0.004) (see Fig. 3B).

### Clinical analyses

To evaluate the impact of the clinical variables for which there was a difference between PG and CP (i.e., positive and negative urgency, depression, anxiety trait, chronic stress and psychiatric comorbidities) on learning strategies, we ran several logistic regressions with the probability of stay in the previous first step choice as dependent variable and type of outcomes and transition as well as the score at the target clinical questionnaire as independent variables. No significant interaction between any of the clinical variables and either reward type or transition x reward type was found (p > 0.05).

## Discussion

The present study aimed at contributing to the understanding of impaired reinforced learning mechanism in behavioral addiction. Based on analysis of choices and reaction times, we found that PG rely less on MB RL prediction while making decision on a two-step task, especially after an unrewarded trial. This finding shed light on potentially important mechanisms involved in inflexible behaviors found in individuals with GD, which are now considered in detail.

Attenuated MB learning signature based on choices was found in PG, with less consideration for transition types, thus leading to fewer rewards. This finding echoes the main idea that impaired MB RL strategy is strongly associated with a symptom dimension comprising compulsive behavior^19^. Further, our results dovetail well with previous studies employing different choice paradigms (e.g., the Fabulous Fruit Task, a reinforcer devaluation test) that found that individuals with drug addiction rely too much on habits instead of goal-directed choices^29^.

In support to the idea of impaired MB control in the clinical sample, we found that PG showed less slowing after rare transitions than CP, which likely reflects reduced MB control^48,49^. Interestingly, the reduction in MB control in PG was particularly important in choices that followed a negative outcome, compared to positive ones. Thus, whereas a negative outcome, in CP signaled the need of additional cognitive control adjustment (MB control) to further avoid these negative outcomes, PG patients failed to recruit these additional control mechanisms. This could occur for a number of possible reasons.

First, the novel finding we provided is that PG is more impulsive than their controls after a non-rewarded trial, as evidenced by faster decisions (expressed as first-stage choice RTs). This phenomenon is in line with previous work reporting that losses (or non-rewarded actions) affect choice by favoring impulsive actions in healthy participants on gambling tasks^50,51^. Our study suggests that impulsive decisions enhances reliance on habits at the detriment of model-based control, possibly due the lack of inhibition of the habit system in the context of frustration. Second, PG could be less sensitive to extinction, a phenomenon characterizing habit formation that can be due to reduced loss aversion^15^, hypersensitivity to rewards, incorrect identification of statistically unlikely sequence of wins as a separate situation from more-commonly experienced losses^52,53^. In line with observed deficits of extinction learning in PG, recent studies suggest that GD could arise from an inflexible association between an action and its reward, even if its outcome is devaluated^52–54^.

Finally, although the illusion of control and uncontrolled cue-dependent relapse are common psychological explanations for behaviours observed in gambling addiction, the nature of the choice paradigm here yields that data too limited to address these possible explanations. Indeed, we failed to find a higher probability in PG than their controls to repeat the previous first step choice after an unrewarded trial, independently of the transition type. Together, those findings support a specific MB deficit in the context of reward expectancies violation, a phenomenon putatively associated with a hyperdopaminergic state^41^ that interferes with inhibition of basal ganglia for which D2 receptors are critical^55,56^. Clearly, additional work is necessary to draw more robust conclusions on neurocognitive determinants of post unrewarded actions that the present work merely suggested. In addition, we found no association between any clinical variables discriminating groups (chronic stress, state and trait anxiety, depression, negative and positive urgency) and the MB signature. This finding indicates that co-occurrence between PG and other psychopathological conditions is not the main reason why PG have goal-directed deficits.

Our findings hold some useful clinical implications. Interestingly, modest clinical outcome (e.g., low remission rate) in the treatment of gambling disorder^57^ could be due to the lack of consideration for the contribution of rudimentary stimulus-response associations to the addictive behavior, in favor of the idea that addiction mainly results from reinforced goal-directed actions (see the self-medication hypothesis)^24^. Because MB RL and cognitive control both involve overcoming habitual, stimulus-driven actions^58^, interventions aimed to improve executive functioning may positively impact on MB contribution. Specifically, electric stimulation (i.e., TDCS) of the dorsolateral prefrontal cortex has been shown to impact a variety of deliberative functions including risk-taking^59^, working memory^60^ and classification learning^61^. Stimulation on the left ventrolateral prefrontal cortex was shown to improve MB control and weight in the decisional balance^62^, but see for negative results^63^. Following this recent effort, further research is needed to test the influence of neurocognitive interventions on MB/MF RL in gambling disorder. In the same way, future studies may examine the usefulness of pharmacological intervention (e.g., amisulpride) blocking D2/D3 receptors to augment the relative contribution of MB learning strategy after a negative outcome. This should be done with careful considerations for other cognitive functions involved in dopamine modulation such as risk taking^64^ and incentive value^65^.

It is worth noting the potential limitations of this study. First, it is possible that the PG group’s behavior is in part attributable to inaccurate expectancies about future events (e.g., the gambling fallacy or hot hand fallacy)^66^. Put differently, inappropriate internal model of the environment’s transition structure could have been responsible for lack of consideration for transitions’ rarity, potentially contributing to both the RT and choice effects. False beliefs about probabilities (e.g., consecutive losses necessary lead to a larger monetary gain or several wins in a row increase the probability of winning later) might lead to suboptimal, yet goal-directed, strategies and, without fully probing participants’ beliefs that takes place during the realization of the task^26,67^, this explanation could not be entirely dismissed. It is therefore possible that decisions considered as habit-like actually result from goal-directed strategies. However, we failed to observe a “hot hand” effect (i.e., the expectation to win after a win) that would have caused faster choice RTs after rewarded trials in PG, in comparison to non-gamblers. Besides, gambling fallacy is more likely after longer runs of losses or wins^68^.

Another potential limitation is that the two-step task does not incentivize participants to use MB control, but instead decouples winnings from the subjects’ choice strategy so as to avoid these variables potentially confounding one another. Interestingly, a recent study reported that MB control can be reliably improved with the provision of larger incentives (e.g., higher stakes) in individuals with several psychiatric conditions^69^. The observed boosting model-based control with larger incentive has been thought to result from on a cost-benefit analysis, that is, higher potential payoffs justify the more effortful decision-making processes (i.e., more model-based control)^58,69,70^. It is worth testing whether the PG deficit in MB RL can be ameliorated in this manner, since a higher sensation seeking trait was both a prominent feature in this population^71^ and a factor associated with greater boosts in MB control in non-clinical participants (83). However, it should be noted that we offered to participants 30 euros plus 10 euros depending on their net performance, which can be considered as very incentive compared to other similar studies.

Finally, the influence of impaired MB learning in the pathogenesis of gambling addiction remains largely unknown. Unlike drug-taking behaviors that may cause profound disruption in learning systems^11^, gambling behaviors offer room to study addiction without the confounding effects of neurotoxicity associated with acute and chronic use of chemical substances^10^. Clearly, in the absence of longitudinal research design, this question cannot be firmly decided. However, a recent preclinical study suggested that individual differences in model-free learning prior to drug use predicted methamphetamine self-administration^72^.

To summarize, we found deficits in learning and decision making in problem gamblers. It is characterized by a reduced MB action control after a negative outcome. This knowledge has highlighted the importance of decision deficits not directly attributable to the neurotoxic effects of chronic drug use.

## Methods

### Participants

Forty-nine individuals with gambling disorder, named problem gamblers (PG), who took part in games involving little skill (i.e., slot machines, video poker, dice and pull tabs), and 33 controls (CP) matched for age and educational level were recruited. All participants were recruited through advertisement and gave written informed consent to be part of the experiment. The experiment was approved by the C.H.U. Brugmann Ethics Committee (n° OM 026) and was performed according to the Declaration of Helsinki.

All participants underwent a semi-structured interview^73^. All PG met the DSM-V criteria^47^ for gambling disorder (range: 3–9) and had a minimum of 8 on the Canadian Problem Gambling Index (CPGI)^74^ (range: 8–27). All PG were active gamblers, and none followed a therapy or treatment. Healthy control subjects had a score of 0 on the CPGI. The exclusion criteria for all participants were the presence of psychotic or neurologic syndromes, antecedents of substance addiction and recent utilization of psychopharmacological substances susceptible to alter cognitive functioning.

The participants’ remuneration was set on 30€ and they were told that they could win up to 10€ more depending on their net performance in the two-step decision task (RL task).

### Questionnaires, experimental tasks and procedure

At the end of the experiment, each participant performed the operation span (OSPAN) task^75^ and filled out clinical questionnaires to estimate substance use, psychological problems and symptoms of psychopathology, current negative emotions, anxiety, depression, stress, impulsivity, craving for gambling. Alcohol use was estimated by the Alcohol Use Disorders Identification Test^76,77^ and nicotine dependence severity by the Fagerström Test for Nicotine Dependence^78^. The psychopathological symptoms were investigated using the total score of the Symptom Checklist-90-Revised (SCL-90-R)^79^. Negative emotions, as well as depression and anxiety, were evaluated by the negative scale of the Positive and Negative Affect Schedule^80^, the short version of the Beck Depression Inventory (BDI)^81^ and the State-Trait Anxiety Inventory (STAI-YA and STAI-YB)^82^, respectively. To measure chronic and current stress, the Social Readjustment Scale (SRRS)^83^ and visual analogue scales (range: 0–10) were administered. Several facets of impulsivity (i.e., negative urgency, positive urgency, lack of premeditation, lack of perseverance and sensation seeking) were evaluated with the short version of the UPPS Impulsive Behavior Scale^84^.

The entirety of the experimental procedure lasted between 1h30 and 2 h and took place individually with two experimented and well-trained neuropsychologists in a quiet room. Upon their arrival, the participants signed an informed consent and filled out a questionnaire about gambling behaviors (CPGI). Prior the RL task, two visual analogic scales (VAS) (i.e., ‘how much do you want to gamble right now?’ and ‘how much do you feel stressed right now?’) were administered. Right after the task, a second series of VAS were given, followed by the remaining clinical questionnaires.

### Two-step decision-making task

**Figure 4:**
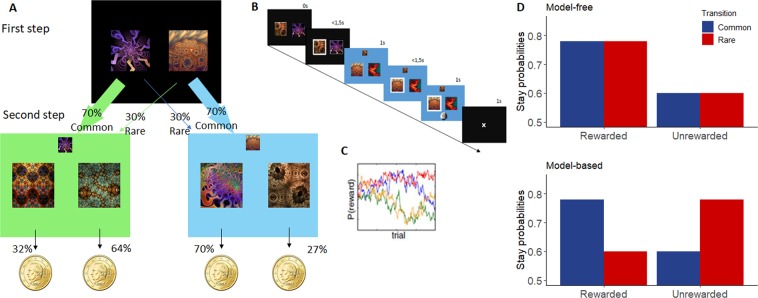
(**A**) Two-step decision task (adapted from Otto *et al*.^85^). (First step) Participants must choose between the two images, leading preferentially to a green or a blue screen, according to fixed probabilities. (Second step) Subject choose between the two images linked to probabilities to win money. Those probabilities slowly change with the time and vary according to the screen color. (**B**) Trial’s design. (**C**) Second step’s changes in probability of reward. (**D**) Theoretical decision pattern according to a pure MF strategy or to a pure MB strategy.

Participants performed 200 trials of two-step decision-making task^43^. This task was divided into two stages (see Fig. 4A). At the beginning of the first step, two fractal images were presented on a black screen, between which the participant had to choose. Each first-stage image led commonly (70%) to one of the two second-stages states and rarely to the other (30%). During the second stage, two images were presented on a green or a blue screen (representing the second-stage ‘state’), between which the participant had to choose. Each image led probabilistically to a reward or not, presented with a visual feedback representing a 10c coins or a 0 during the 1-second feedback interval. In order to assure continual learning and exploration during the task, each second-step image’s probability to reward money slowly varied during the task according to Gaussian random walks (*SD* = 0.025). They had 3 second to perform each choice and the inter-stage and inter-trial intervals both lasted 1 second.

Prior to the task, participants were given extensive instructions about the task’s structure^19^. They were instructed that the first choice would preferentially lead towards a blue or a green screen, each one associated with different probabilities of winning, and that their choice at the second screen would depend on their choice on the first screen. It was stressed that transition probabilities between the first and the second stage would be constant while the probabilities of winning at the second stage could vary over time. Participants then completed a tutorial and had to provide correct responses to a quiz including three questions about the task’s structure^19^. In case of incorrect response to any of them, the explanation phase took place again. They sat in front of a laptop with an AZERTY keyboard. The letter ‘E’ was assigned to the left image and the letter ‘I’ to the right image.

Several measures were considered: The outcome of each second-stage choice (reward or not), transition type (common or rare), the response times to rewarded or unsuccessful trials on frequent or rare transitions, and the probabilities of making two consecutive identical first-stage choices according to the type of transition and reward (termed *p*(stay)). A pure model-free strategy predicts purely reinforcement-guided choices: a repetition of the previous trial’s first-stage choice only when it was previously rewarded, and a shift occurring after a previous trial being not rewarded. A pure MB strategy takes the task structure and transition type into consideration and predicts a repetition of the previous trial’s first step only if it was rewarded and following a common transition or if it was not rewarded after a rare transition (see Fig. 4D).

### Data analyses

All analyses were performed using IBM SPSS Statistics v25 and RStudio Version 1.1.456. To ensure that participants’ data reflected a sufficient level of engagement to the task, in the same way as a previous study^85^, those who repeated previously rewarded second-step responses at a rate less than 50%, those who did not answer before the deadline more than 20 times, and those who did not try every image in each stage were removed from the data analyses. This resulted in the removement of 4 subjects. Groups were compared on each clinical variable (e.g., depression, anxiety, impulsivity, stress) by using t-tests or non-parametric tests, where appropriate.

A mixed logistic regression was carried out to analyze the influence of group (PG, CP), of previous transition type (common, rare) and of previous outcome (reward, no reward) on the probability to maintain a previous trial first step choice (stay, switch). As MB and MF learning predicting distinct patterns of first-stage repetitions to the previous trial’s events (reward and transition type), this analysis allowed for a quantitative evaluation of their contribution to the trial-by-trial learning. A pure MF strategy rends the first stage choice only impacted by the previous trial’s outcome, independently of the previous trial’s transition type, thus predicting only a main effect of the outcome. On the other hand, a pure MB strategy predicts an interaction between the outcome and the transition type^85^. Secondly, in order to test our hypothesis that PG had a more pronounced MB impairment after unrewarded trials, we performed two more logistic regressions, separately examining trials following a reward and trials following the absence of reward.

To assess further decision strategies based on reaction times, a mixed ANOVA with the current trial’s transition type (rare, common) as within-factor in PG and CP as between-factor was performed on the second stage response time. Indeed, the difference between second-stage RTs after common versus rare transitions reflects the level of involvement of MB control^48,49^.

To examine the influence of clinical status other than gambling disorder on decisional strategy on the two-step task, each clinical variable that discriminate the two groups was added separately to the mixed logistic regression.

## Data Availability

All data will be made available on the following lab website: http://psymed.ulb.be/.
